# Inhibition of Melanogenesis *Versus* Antioxidant Properties of Essential Oil Extracted from Leaves of *Vitex negundo* Linn and Chemical Composition Analysis by GC-MS

**DOI:** 10.3390/molecules17043902

**Published:** 2012-03-30

**Authors:** Huey-Chun Huang, Tzu-Yun Chang, Long-Zen Chang, Hsiao-Fen Wang, Kuang-Hway Yih, Wan-Yu Hsieh, Tsong-Min Chang

**Affiliations:** 1 Department of Medical Laboratory Science and Biotechnology, China Medical University, No. 91 Hsueh-Shih Road, Taichung City 40402, Taiwan; Email: lchuang@mail.cmu.edu.tw; 2 Department of Applied Cosmetology & Master Program of Cosmetic Science, Hung Kuang University, No. 34, Chung-Chie Rd., Shalu, Taichung City 43302, Taiwan; Email: sunmidi100678@yahoo.com.tw (T.-Y.C.); khyih@sunrise.hk.edu.tw (K.-H.Y.); opency.tw@gmail.com (W.-Y.H.); 3 General of Agriculture Bureau of Taichung City, Taichung, Taiwan, No. 89, Sec 2, Taichung Port Rd., Xitun Dist., Taichung City 40701, Taiwan; Email: m35208@taichung.gov.tw; 4 Department of Hair Styling & Design, Hung Kuang University, No. 34, Chung-Chie Rd., Shalu, Taichung City 43302, Taiwan; Email: ygl615@yahoo.com.tw

**Keywords:** *Vitex negundo *Linn, essential oil, tyrosinase, melanin, antioxidant

## Abstract

This study was aimed at investigating the antimelanogenic and antioxidative properties of the essential oil extracted from leaves of* V. negundo* Linn and the analysis of the chemical composition of this essential oil. The efficacy of the essential oil was evaluated spectrophotometrically, whereas the volatile chemical compounds in the essential oil were analyzed by gas chromatography-mass spectrometry (GC-MS). The results revealed that the essential oil effectively suppresses murine B16F10 tyrosinase activity and decreases the amount of melanin in a dose-dependent manner. Additionally, the essential oil significantly scavenged 2,2-diphenyl-1-picrylhydrazyl (DPPH) and 2,2'-azino-bis(3-ethylbenzthiazoline-6-sulphonic acid) (ABTS) radicals, and showed potent reducing power *versus* metal-ion chelating properties in a dose-dependent pattern. The chemical constituents in the essential oil are sesquiterpenes (44.41%), monoterpenes (19.25%), esters (14.77%), alcohols (8.53%), aromatic compound (5.90%), ketone (4.96%), ethers (0.4%) that together account for 98.22% of its chemical composition. It is predicted that the aromatic compound in the essential oil may contribute to its antioxidant activities. The results indicated that essential oil extracted from *V. negundo* Linn leaves decreased melanin production in B16F10 melanoma cells and showed potent antioxidant activities. The essential oil can thereby serve as an inhibitor of melanin synthesis and could also act as a natural antioxidant.

## 1. Introduction

Melanin is responsible for skin color and plays a key role in protecting the skin from UV light-induced damage. Tyrosinase is the rate-limiting enzyme in the first two steps of the melanin biosynthesis pathway, in which L-tyrosine is hydroxylated to L-DOPA, and L-DOPA is further oxidized into the corresponding *ο*-quinone [1]. It is found that some skin disorders such as melasma, freckles, age spots and other hyperpigmentation syndromes are the result of abnormal accumulation of melanin [2]. Therefore, a number of skin depigmenting chemicals such as kojic acid [3] and arbutin [4] have been applied as skin whitening cosmetics for prevention or treatment of undesirable skin pigmentation [5]. 

Free radicals and reactive oxygen species (ROS) are considered to be associated with some diseases such as inflammation [6], aging and age-related diseases [7]. Antioxidants can interfere with oxidation processes by acting as free radical scavengers, ROS scavengers or by chelating oxidation-catalytic metals [8,9]. Numerous natural antioxidants or commercial antioxidant supplements have been used to reduce oxidative damage in the human body [10]. However, some synthetic antioxidants such as *tert*-butyl hydroxytoluene (BHT) and *tert*-butyl hydroxyanisole (BHA) were reported to show carcinogenic effects in humans [11], hence a number of reports on plant-derived antioxidants have appeared during the past decade. Moreover, melanogenesis has been reported to produce hydrogen peroxide (H_2_O_2_) and other ROS which place melanocytes under high-grade oxidative stress. Interestingly, it is well known that several ROS scavengers and inhibitors of ROS generation may downregulate UV-induced melanogenesis [12]. Therefore, melanogenesis inhibitors, antioxidants and ROS scavengers have been increasingly added to skin care cosmetics for the prevention of hyperpigmentation [13]. 

*Vitex negundo* Linn (Verbenaceae) is a large aromatic shrub found throughout India, east Africa, the Philippine islands, Malaysia and also found mainly in the warm zones in Taiwan. It has been extensively studied for its various pharmacological properties including antinociceptive [14], anti-convulsant [15] and anti-inflammatory [16] activities. Most importantly, the extracts from leaves and roots of* V. negundo* Linn have been applied in medicine. For example, water extract of fresh leaves of* V. negundo* Linn is reported to show analgesic, anti-inflammatory and anti-histaminic properties [17]. Recently, the methanol extract of the roots of *V. negundo* Linn has been reported to exhibit inhibitory effects on mushroom tyrosinase [18]. However, to the best of our knowledge, there is no report about application of essential oil extracted from leaves of *V. negundo* Linn for potential use in skin care cosmetics. In the present study, we aimed at investigating the potential skin whitening efficacy and antioxidant activity of essential oil extracted from leaves of *V. negundo* Linn and to analyze the chemical constituents of the essential oil by GC-MS. Hence, antimelanogenic *versus* antioxidant efficacy of the essential oil and its chemical composition are reported in the study. 

## 2. Results and Discussion

### 2.1. Inhibition of Melanogenesis by *V. negundo* Linn Essential Oil

#### 2.1.1. Inhibitory Effect of *V. negundo* Linn Essential Oil on Mushroom Tyrosinase Activity

**Figure 1:**
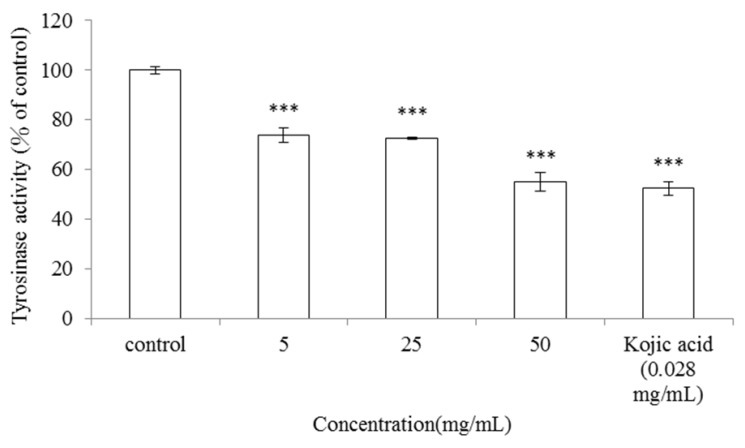
Inhibitory effect of *V. negundo *Linn essential oil on mushroom tyrosinase activity. Different concentrations of *V. negundo *Linn essential oil (5–50 mg/mL) or kojic acid (0.028 mg/mL) were incubated with the same units of mushroom tyrosinase. Results are represented as percentages of control, and data are presented as mean ± SD for three separate experiments. Values are significantly different by comparison with control. *** *p* < 0.001.

Tyrosinase plays an essential role in the first two steps of melanin synthesis pathway. It converts L-tyrosine to L-DOPA and oxidizes L-DOPA to form dopachrome. Mushroom tyrosinase is widely used as the target enzyme in screening potential inhibitors of melanogenesis. In order to assay the inhibitory effect of the *V. negundo *Linn essential oil on mushroom tyrosinase activity, dose-dependent inhibition experiments were carried out in triplicate. The results indicated that mushroom tyrosinase activity was inhibited by the various tested concentrations of *V. negundo *Linn essential oil (5, 25 and 50 mg/mL) in a dose-dependent manner. The residual tyrosinase activity was 73.94 ± 2.89%, 72.72 ± 0.38% and 55.22 ± 3.73% of control, respectively (*p* < 0.001). Kojic acid was used as a positive standard in this assay. The mushroom tyrosinase activity was also inhibited by kojic acid (0.028 mg/mL) and the observed enzyme activity was 52.41 ± 2.82% of that of control (*p* < 0.001) (Figure 1). 

The results indicated that 50 mg/mL of* V. negundo *Linn essential oil show a similar inhibitory effect on mushroom tyrosinase activity as kojic acid, hence essential oil extracted from leaves of *V. negundo *Linn may act as a potential tyrosinase inhibitor.

#### 2.1.2. Effect of *V. negundo* Linn Essential Oil on Melanin Synthesis in B16F10 Cells

**Figure 2:**
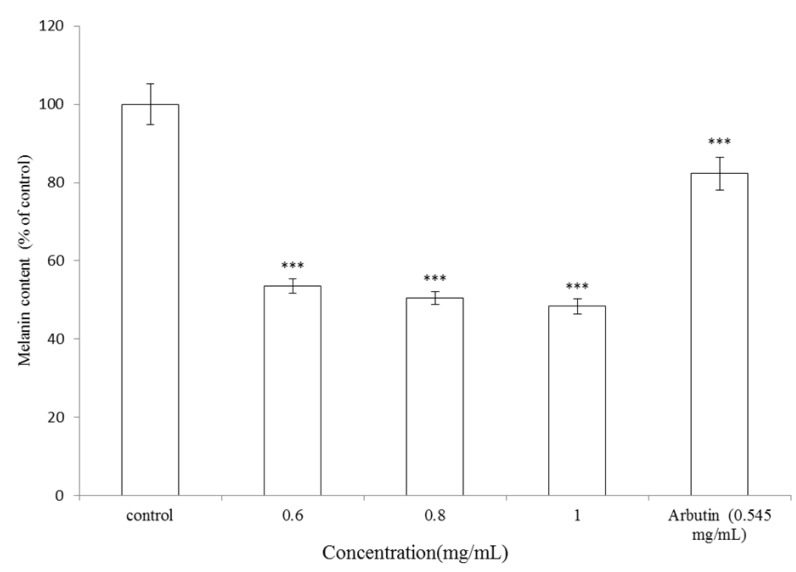
Effect of *V. negundo *Linn essential oil on melanin synthesis in B16F10 cells. Melanin content assessment was performed as briefly described below. The cells were cultured with α-MSH (100 nM) for 24 h, and then treated with various concentrations of *V. negundo *Linn essential oil (0.6–1.0 mg/mL) or arbutin (0.545 mg/mL) for 24 h. Results are represented as percentages of control, and data are presented as mean ± SD for three separate experiments. Values are significantly different by comparison with control. *** *p* < 0.001.

To determine the antimelanogenic activity of *V. negundo *Linn essential oil, the inhibitory effect of the essential oil on melanin content in B16F10 melanoma cells was assayed. B16F10 cells were stimulated with α-MSH (α-melanocyte stimulating hormone) and cultured in the presence of the essential oil at 0.6, 0.8 and 1.0 mg/mL or arbutin (0.545 mg/mL). Treatment with *V. negundo *Linn essential oil showed a significant inhibitory effect on melanin synthesis in a dose-dependent manner. The melanin content was represented as percentage of control. After treatment, the melanin content was 53.6 ± 1.85%, 50.53 ± 1.58% and 48.39 ± 1.89% for 0.6, 0.8 and 1.0 mg/mL of the essential oil, respectively (*p* < 0.001). IC_50_ of the essential oil is 0.86 mg/mL. Meanwhile, B16F10 cells were treated with arbutin (0.545 mg/mL) as positive standard, and the remained intracellular melanin content was 82.34 ± 4.18% of control for arbutin (*p* < 0.001) (Figure 2). The results shown in Figure 2 indicated that essential oil extracted from leaves of* V. negundo *Linn present a stronger inhibitory effect on melanin formation in B16F10 cells than arbutin.

#### 2.1.3. Effects of *V. negundo* Linn Essential Oil on Tyrosinase Activity in B16F10 Cells

**Figure 3:**
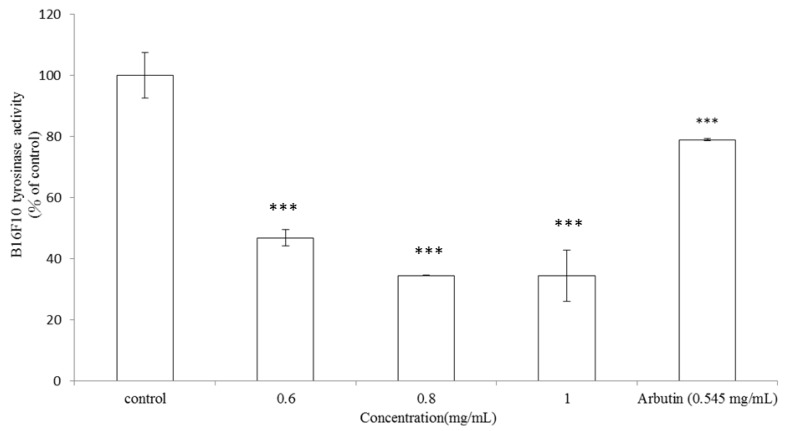
Effects of *V. negundo *Linn essential oil on tyrosinase activity in B16F10 cells. Enzyme assay was performed as previously described. Briefly, B16F10 melanoma cells were stimulated with α-MSH (100 nM) for 24 h, and treated with *V. negundo *Linn essential oil (0.6–1.0 mg/mL) or arbutin (0.545 mg/mL). Results are represented as percentages of control, and the data are mean ± SD for three separate experiments. Values are significantly different by comparison with control. *** *p* < 0.001.

To examine more precisely the mechanism of action of *V. negundo *Linn essential oil on melanogenesis, we assessed intracellular tyrosinase activity in B16F10 melanoma cells. The cells were stimulated with α-MSH (100 nM) for 24 h, and with various concentrations of the essential oil at 0.6, 0.8 and 1.0 mg/mL or arbutin (0.545 mg/mL) for another 24 h. The *V. negundo *Linn essential oil significantly inhibited α-MSH-induced tyrosinase activity in a dose-dependent pattern (Figure 3). After these treatments, the observed intracellular tyrosinase activity was 46.82 ± 2.71%, 34.53 ± 0.22% and 34.46 ± 8.41% for 0.6, 0.8 and 1.0 mg/mL of the essential oil, respectively (*p* < 0.001). IC_50_ of the essential oil is 0.43 mg/mL. Meanwhile, the intracellular tyrosinase activity was 78.88 ± 0.33% after the cells were treated with arbutin (0.545 mg/mL) (*p* < 0.001). The results shown in Figure 3 were in accordance with the results indicated in Figure 2, which means that essential oil extracted from leaves of *V. negundo *Linn inhibited B16F10 intracellular tyrosinase activity and then decreased the melanin content in a dose-dependent manner.

It has been reported that eight lignans isolated from the methanol extract of the roots of *V. negundo *Linn show inhibitory effects on mushroom tyrosinase activity [19]. However, the authors did not determine the effects of those lignans on B16F10 intracellular tyrosinase activity or melanin content. Most importantly, there are no reports about dermatological applications of essential oil extracted from leaves of* V. negundo *Linn. Furthermore, the present study proved that the essential oil shows considerable whitening potential in the B16F10 analytical assays. The results shown in Figure 2 indicate that* V. negundo *Linn essential oil has a stronger inhibitory effect on melanin formation in B16F10 cells than arbutin does. Additionally, the essential oil also inhibited α-MSH-induced intracellular tyrosinase activity in a dose-dependent pattern (Figure 3). 

In these experiments, α-MSH acted as a cAMP inducer to stimulate melanin synthesis. It is evidenced that α-MSH can bind melanocortin 1 receptor (MC1R) and activate adenylate cyclase, which in turn catalyzes ATP to cAMP and increases intracellular cAMP level [20]. In this report, the results show that* V. negundo *Linn essential oil inhibited melanogenesis induced by α-MSH mediated intracellular cAMP up-regulation.

### 2.2. Antioxidant Capacity of *V. negundo* Linn Essential Oil

#### 2.2.1. DPPH Assay

**Figure 4:**
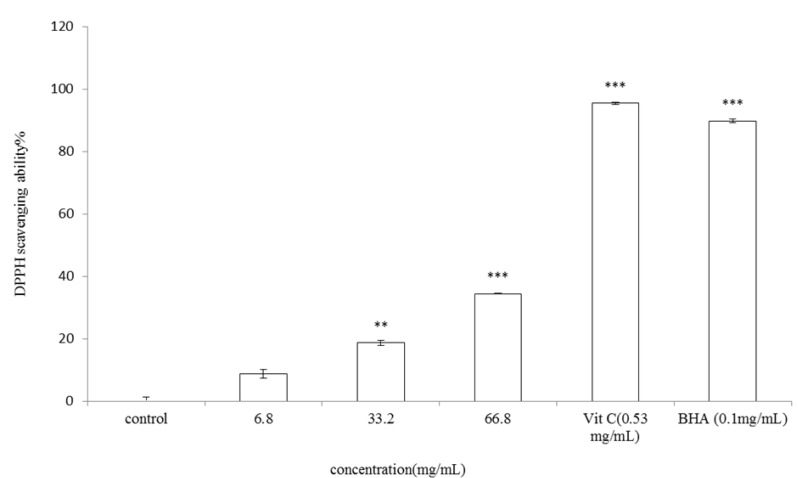
Scavenging effects of *V. negundo *Linn essential oil on DPPH radical.The essential oil at various concentrations (final concentration 6.8, 33.2, 66.8 mg/mL), vitamin C (0.53 mg/mL) and BHA (0.1 mg/mL) were interacted with DPPH. Results are represented as percentages of control, and the data are mean ± SD for three separate experiments. Values are significantly different by comparison with control. ** *p* < 0.01, *** *p* < 0.001.

The DPPH assay is known to provide reliable information concerning the antioxidant capacity of specific compounds or extracts across a short time scale. The antioxidant activity of *V. negundo *Linn essential oil was first determined by measuring the DPPH scavenging ability. The* V. negundo *Linn essential oil shows DPPH radicals scavenging activity in a dose-dependent manner as shown in Figure 4. DPPH scavenging activity of 6.8, 33.2 and 66.8 mg/mL of the essential oil was 8.67 ± 1.43%, 18.62 ± 0.72% (*p* < 0.01) and 34.41 ± 0.07% (*p* < 0.001) of control, respectively. IC_50_ of the essential oil is 103.85 mg/mL. However, the activities were less effective than those of vitamin C (95.43 ± 0.40%) and BHA (89.75 ± 0.58%) (*p* < 0.001).

#### 2.2.2. ABTS Assay

**Figure 5:**
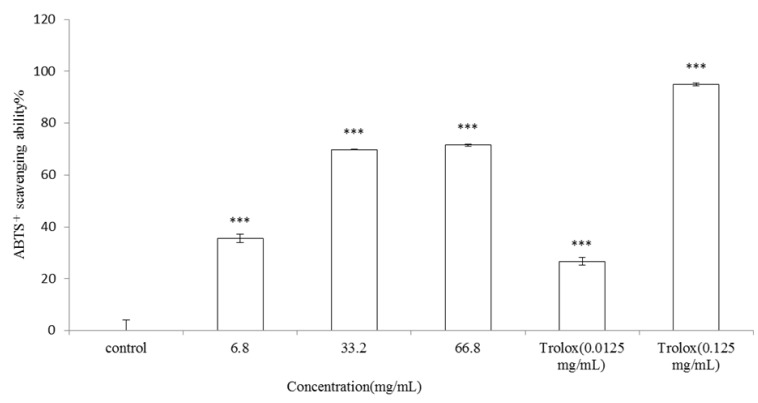
ABTS^+^ radical scavenging effects of *V. negundo *Linn essential oil.The essential oil (final concentration 6.8, 33.2, 66.8 mg/mL) or Trolox^®^ (0.0125 or 0.125 mg/mL) were mixed with ABTS. Results are represented as percentages of control, and the data are mean ± SD for three separate experiments. Values are significantly different by comparison with control. *** *p* < 0.001.

The ABTS assay was employed to measure the antioxidant activity of the essential oil. Different concentrations of the* V. negundo *Linn essential oil (final concentration 6.8, 33.2, 66.8 mg/mL) or Trolox^®^ (0.0125 or 0.125 mg/mL) were incubated with ABTS^+^ solution. The ABTS^+^ scavenging capacity of the essential oil was 35.62 ± 1.69%, 69.81 ± 0.06% and 71.61 ± 0.42% of control for the essential oil at the dosage of 6.8, 33.2 and 66.8 mg/mL, respectively (*p* < 0.001). IC_50_ of the essential oil is 19.94 mg/mL. Meanwhile, the ABTS^+^ scavenging capacity of Trolox^®^ (0.0125 mg/mL) was 26.68 ± 1.50% (*p* < 0.001). The results indicated that essential oil extracted from the leaves of *V. negundo *Linn scavenges ABTS^+^ free radical significantly in a dose-dependent manner. However, the higher concentration of Trolox^®^ (0.125 mg/mL) showed the strongest ABTS^+^ radical scavenging capacity (94.97 ± 0.49%; *p* < 0.001; Figure 5).

#### 2.2.3. Determination of Reducing Power of *V. negundo* Linn Essential Oil

**Figure 6:**
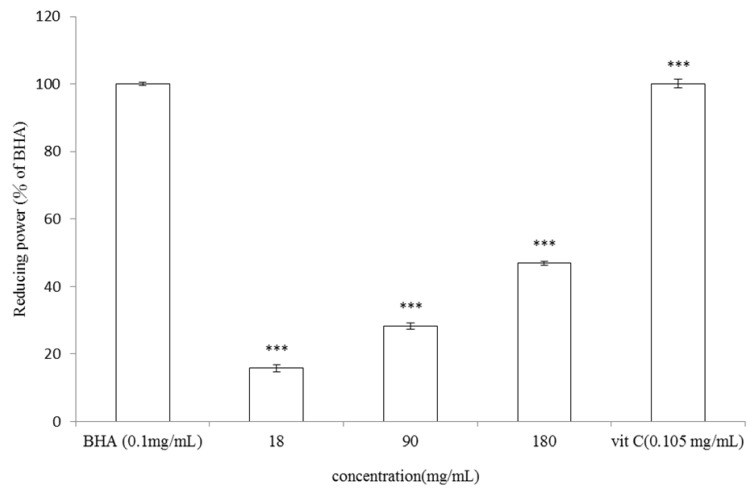
Reducing power of *V. negundo *Linn essential oil. Different concentrations of* V. negundo *Linn essential oil (18, 90, 180 mg/mL), vitamin C (0.105 mg/mL) or BHA (0.1 mg/mL) were used in the study. Results are represented as percentages of control, and the data are mean ± SD for three separate experiments. Values are significantly different by comparison with control. *** *p* < 0.001.

To determine the reducing power of essential oil extracted from leaves of *V. negundo *Linn, various concentrations of the essential oil or vitamin C (0.105 mg/mL) or BHA (0.1 mg/mL) were tested. The results shown in Figure 6 revealed that higher concentrations of *V. negundo *Linn essential oil displayed apparent reducing power. The reducing power of 18, 90, 180 mg/mL of *V. negundo *Linn essential oil were 15.8 ± 0.99%, 28.35 ± 0.93% and 46.87 ± 0.52% when compared to 0.1 mg/mL of BHA (*p* < 0.001). Even when the dosage was increased, the reducing power of the essential oil was still lower than those of vitamin C or BHA, which were equivalent to each other. 

#### 2.2.4. Metal-Ion Chelating Activity of *V. negundo* Linn Essential Oil

**Figure 7:**
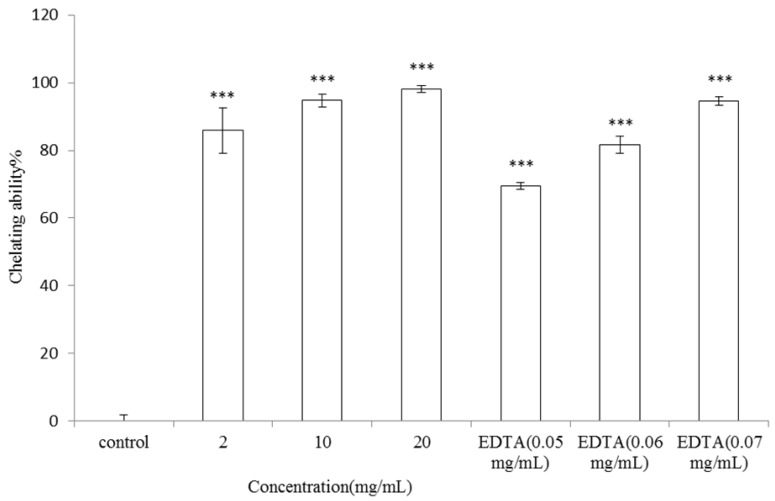
Metal-ion chelating activity of *V. negundo *Linn essential oil. Different concentrations of* V. negundo *Linn essential oil (2, 10, 20 mg/mL) or EDTA (0.05–0.07 mg/mL) were used in the study. Results are represented as percentages of control, and the data are mean ± SD for three separate experiments. Values are significantly different by comparison with control. *** *p* < 0.001.

Antioxidants may form insoluble metal complexes with ferrous ion and then inhibit interaction between metal and lipid. The metal-ion chelating abilities of 2, 10, and 20 mg/mL of* V. negundo *Linn essential oil were 85.89 ± 6.75%, 94.71 ± 1.96% and 98.12 ± 1.11% of control, respectively (*p* < 0.001). On the other hand, the metal-ion chelating capacities of 0.05, 0.06 and 0.07 mg/mL of ethylene diamine-*N,N*-tetraacetic acid (EDTA) were 69.51 ± 1.09%, 81.63 ± 2.53% and 94.56 ± 1.31%, respectively (*p* < 0.001; Figure 7). The results shown in Figure 7 indicated the potent antioxidant capacity of the essential oil extracted from the leaves of *V. negundo *Linn.

Human skin is a preferred target of oxidative stress induced by UV exposure and environmental oxidizing pollutants. It is reported that ultraviolet irradiation induces the formation of ROS in cutaneous tissue provoking toxic changes such as lipid peroxidation and enzyme inactivation [20]. To counteract this oxidative damage, the skin is equipped with a network of enzymatic and non-enzymatic antioxidant systems. To elucidate the antioxidant activity of *V. negundo *Linn essential oil, DPPH, ABTS^+^ radical scavenging activity, reducing power and metal-chelating capacity of the essential oil was determined as described previously. *V. negundo *Linn essential oil showed considerable antioxidant potential in all the analytical studies. The results displayed a dose-dependent increase in antioxidant potential over different ranges with distinct efficiencies. The differential radical scavenging activities of the essential oil against DPPH and ABTS^+^ radicals may be referred to the different mechanisms of the antioxidant-radical reactions in the two assays. Besides, the stoichiometry of reactions between the potential antioxidant compounds in the essential oil may be different, which result in the difference in radical scavenaging capacity [21]. The reducing power of a Fe^3+^/ferricyanide complex to the ferrous form can serve as an indicator of the antioxidant ability. The existence of reductones accounts for the reducing power and exhibit the antioxidant activities by donating a hydrogen atom. The percentage of ketone content in the essential oil is only 4.96% which may account for the lower reducing power of the essential oil. Antioxidants may form insoluble metal complexes with ferrous ion and then inhibit interaction between metal and lipid. The higher metal-ion chelating capacity of the essential oil indicated its potent antioxidant activity (Figure 7). 

### 2.3. Chemical Composition of *V. negundo* Linn Essential Oil

The chemical composition of* V. negundo *Linn essential oil was analyzed by GC-MS with the results shown in Table 1. The major components in the oil are sesquiterpenes (44.41%) which consist of caryophyllene (18.27%), eremophilene (12.76%), caryophyllene oxide (11.33%), *β*-bisabolene (0.94%), humulene (0.58%) and *α*-bergamotene (0.53%). There is a 19.25% of monoterpenes in the oil, including sabinene (19.04%) and 1(*R*)-*α*-pinene (0.21%). The oil contains four types of esters, namely *β*-terpinyl acetate (8.99%), linalyl formate (3.72%), nerol acetate (1.18%) and geranyl acetate (0.88%). Linalool (4.27%), (−)-menthol (1.44%) and (−)-terpinen-4-ol (2.82%) are the three alcohols found in the oil. The only aromatic compound and ketone found in the oil were *o*-cymene (5.90%) and menthone (4.96%), respectively. Eucalyptol (0.40%) is a kind of ether in the oil. However, there are also 1.78% of unknown compounds in the oil. 

The GC-MS data thus revealed the presence of seven types of chemical components, which are summarized in Table 1, including sesquiterpenes (44.41%), monoterpenes (19.25%), esters (14.77%), alcohols (8.53%), aromatic compound (5.90%), ketone (4.96%), ether (0.4%) and together they account for 98.22% of the essential oil. An earlier analysis of essential oil from *V. negundo *leaf reported that it contained *β*-caryophyllene, caryophyllene oxide, globulol, viridiflorol and sabinene as major constituents [22].

**Table 1:** Chemical composition of essential oil from leaves of* V. negundo* Linn. The chemical constituents of *V. negundo* Linn essential oil was analyzed by a Thermo GC-MS system.

R_t_^a^	Compound ^b^	M. f. ^c^	Peak area (%)	Classification
23.99	Sabinene	C_10_H_16_	19.04	monoterpene
26.80	Eucalyptol	C_10_H_17_O	0.40	ether
27.25	*o*-Cymene	C_10_H_14_	5.90	aromatic compound
27.47	*β*-Terpinyl acetate	C_12_H_20_O_2_	8.99	ester
28.18	1R- *α*-Pinene	C_10_H_16_	0.21	monoterpene
30.94	Linalool	C_10_H_18_O	4.27	alcohol
33.03	Menthone	C_10_H_18_O	4.96	ketone
33.97	(−)-Menthol	C_10_H_19_O	1.44	alcohol
34.15	(−)-Terpinen-4-ol	C_10_H_19_O	2.82	alcohol
39.07	Linalyl formate	C_11_H_18_O_2_	3.72	ester
46.54	Nerol acetate	C_12_H_20_O_2_	1.18	ester
47.46	Geranyl acetate	C_11_H_20_O_2_	0.88	ester
48.83	Caryophyllene	C_15_H_24_	18.27	Sesquiterpene
49.54	*α*-Bergamotene	C_15_H_24_	0.53	Sesquiterpene
50.13	Humulene	C_15_H_24_	0.58	Sesquiterpene
51.37	Eremophilene	C_15_H_24_	12.76	Sesquiterpene
52.11	*β*-Bisabolene	C_15_H_24_	0.94	Sesquiterpene
54.31	Caryophyllene oxide	C_15_H_24_O	11.33	Sesquiterpene
	Unknown		1.78	

^a^ R_t_: Retention time (min); ^b^ The components were identified by their mass spectra and retention indices (RIs) with that of the Wiley and NIST mass spectral databases and the previously published RIs; ^c^ M. f.: Molecular formula.

The results in the present study matches the results of earlier findings except in that globulol and viridiflorol were not found in our studies. There are several factors involved in the regulation of the constituents in the essential oil such as different plant cultivation and/or harvesting procedures. Furthermore, different analytical technique may also result in different GC-MS data. The only aromatic compound, namely *o*-cymene (5.90%), may account for the antioxidant properties of the essential oil. Additionally, the other non-aromatic compounds such as sesquiterpenes or monoterpenes may also contribute towards the antioxidant activities of the essential oil extracted from the leaves of* V. negundo *Linn.

## 3. Experimental

### 3.1. Plant Material and Isolation of Essential Oils

The leaves of *V. negundo *Linn were collected during July to September in 2010 and identified at the Taichung District Agricultural Research and Extension Station in Taiwan. Essential oil was isolated by hydrodistillation of the fresh leaves (2 kg) in a Clevenger-type apparatus at 100 °C for 2 h. The essential oil was collected in a sealed glass bottle and stored at 4 °C in a refrigerator until analysis. In the following experiments, the essential oil was diluted with dimethyl sulfoxide (DMSO) and DMSO was used as a negative control. The IC_50_ is the concentration of the essential oil where the absorbance is reduced by half.

### 3.2. Mushroom Tyrosinase Actvity Assay

To assay the potential inhibitory effects of *V. negundo *Linn essential oil on DOPA-oxidase activity of mushroom tyrosinase, dose-dependent inhibition experiments were carried out in triplicate as described previously with a slight modification [23]. Briefly, aqueous solution of mushroom tyrosinase (20 μL, 200 units) was added to a 96-well microplate, in a total volume of 200 μL mixture containing 5 mM L-DOPA dissolved in 50 mM phosphate buffer (pH 6.8) and *V. negundo *Linn essential oil (5, 25, 50 mg/mL) or kojic acid (0.028 mg/mL). The assay mixture was incubated at 37 °C for 30 min. Following incubation, the amount of dopachrome produced in the reaction mixture was determined by spectrophotometric analysis of absorbance at 490 nm.

### 3.3. Intracellular Melanin Content Measurement

B16F10 melanoma cells (ATCC CRL-6475) were cultured in DMEM with 10% fetal bovine serum (FBS; Gibco, NY, USA) and penicillin/streptomycin (100 IU/50 μg/mL) in a humidified atmosphere containing 5% CO_2_ in air at 37 °C. Intracellular melanin content was measured as previous described by Tsubol *et al*. with some modifications [24]. The cells were treated with α-MSH (100 nM) for 24 h, and further treated with either* V. negundo *Linn essential oil (final concentration 0.6, 0.8, 1.0 mg/mL) or arbutin (0.545 mg/mL) for another 24 h. After treatments, the cells were detached by incubation in trypsin/EDTA and subsequently centrifuged at 5,000 g for 5 min, and then the cell pellets were solubilized in 1 N NaOH at 60 °C for 60 min. The melanin content was assayed at 405 nm absorbance by spectrophotometric analysis.

### 3.4. Intracellular Tyrosinase Activity Assay

B16F10 intracellular DOPA-oxidase activity of tyrosinase was determined as described previously with minor modifications [25]. Briefly, the cells were treated with α-MSH (100 nM) for 24 h, and then further treated with various concentrations of* V. negundo *Linn essential oil (final concentration 0.6, 0.8, 1.0 mg/mL) or arbutin (0.545 mg/mL) for another 24 h. After treatments, the cells were washed twice with phosphate-buffered saline and homogenized with 50 mM PBS (pH 7.5) buffer containing 1.0% Triton X-100 and 0.1 mM PMSF (phenylmethylsulfonyl fluoride; a serine proteinase inhibitor). Cellular extracts (100 μL) were mixed with freshly prepared L-DOPA solution (0.1% in phosphate-buffered saline) and incubated at 37 °C for 30 min. The absorbance at 490 nm was measured with a microplate reader Gen 5^TM^ (BIO-TEK Instrument, Winooski, VT, USA) to monitor the production of dopachrome.

### 3.5. DPPH Scavenging Activity Assay

The antioxidant activity of *V. negundo *Linn essential oil was first determined by measuring the DPPH scavenging ability [26] as modified by [27]. The essential oil at various concentrations (final concentration 6.8, 33.2, 66.8 mg/mL) was added to DPPH (60 μM, 2.9 mL) solution. When DPPH reacts with an antioxidant that can donate hydrogen, it is reduced and the resulting decrease in absorbance at 517 nm was recorded using a UV-Vis spectrophotometer (Jasco, V-630, Tokyo, Japan). In this study, vitamin C (0.53 mg/mL) and BHA (0.1 mg/mL) were used as antioxidant standards.

### 3.6. ABTS^+^ Scavenging Capacity Assay

The ABTS decolorisation assays were carried out as previously described [28] and it involves the generation of ABTS^+^ chromophore by oxidation of ABTS with potassium persulfate. The ABTS radical cation (ABTS^+^) was produced by reacting 7 mM stock solution of ABTS with 2.45 mM potassium persulfate and allowing the mixture to stand in the dark for at least 6 h before use. Absorbance at 734 nm was measured 10 min after mixing of different concentrations of the* V. negundo *Linn essential oil (final concentration 6.8, 33.2, 66.8 mg/mL) with 1 mL of ABTS^+^ solution. The ABTS^+^ scavenging capacity of* V. negundo *Linn essential oil was compared with that of Trolox^®^ (0.0125 or 0.125 mg/mL).

### 3.7. Determination of Reducing Power

The reducing power of the essential oil was determined according to the method of Oyaizu [29]. Different concentrations of* V. negundo *Linn essential oil (18, 90, 180 mg/mL), vitamin C (0.105 mg/mL) or BHA (0.1 mg/mL) was mixed with phosphate buffer (2.5 mL, 0.2 M, pH 6.6) and potassium ferricyanide [K_3_Fe(CN)_6_] (2.5 mL, 1% w/v). The mixture was incubated at 50 °C for 20 min. A portion (2.5 mL) of trichloroacetic acid (10% w/v) was added to the mixture, which was then centrifuged at 1,000 g for 10 min. The upper layer of solution (2.5 mL) was mixed with distilled water (2.5 mL) and FeCl_3_ (0.5 mL, 0.1% w/v), and the absorbance was measured at 700 nm in a UV-Vis spectrophotometer. Higher absorbance of the reaction mixture indicated greater reducing power of the test sample.

### 3.8. Measurement of Metal-Ion Chelating Capacity

The chelation of ferrous ions by the *V. negundo *Linn essential oil or EDTA was determined by the method of [30] with slight modifications. Different concentrations of essential oil (2, 10, 20 mg/mL) were added to a solution of 1 mM FeCl_2_ (0.05 mL). Then 0.1 mL of ferrozine (1 mM) was added to the reaction mixture and the mixture was quantified to 1 mL with methanol, left standing at 25 °C for 10 min. The absorbance of the reaction mixture was measured at 562 nm. The percentage of chelating ability was calculated as follows:

                chelating ability % = [(A_1_− A_2_)/A_1_ × 100]

where A_1_ is the absorbance of control and A_2_ is the absorbance in the presence of essential oil or EDTA.

### 3.9. Gas Chromatography-Mass Spectrometry (GC-MS)

The volatile chemical compounds in the *V. negundo *Linn essential oil were analyzed using a Thermo GC-MS system (GC-MS Trace DSQ-Mass Spectrometer, MSD 201351, Thermo, Minneapolis, MN, USA). An Equity^TM-5^ capillary column (Supelco, St. Louis, MO, USA) with 30 m length and 0.25 mm inside diameter with a 0.25 μm thick film was used. The oven temperature gradient was programmed as follows: isothermal at 40 °C, followed by a 5 °C temperature ramp every minute to 100 °C, which was held for 5 min. Subsequently, the temperature was increased 5 °C every minute to 250 °C and held for 20 min. The carrier gas was helium (1 mL/min). The injection port’s and detector’s temperature were 250 °C. Ionization of the test essential oil (1 μL) was performed in the EI mode (70 eV). The linear retention indices for all compounds were determined by co-injection of the essential oil with a solution containing a homologous series of C_8_–C_22_ *n*-alkanes [31]. The individual components were identified by retention indices and compared with compounds known from the literature [32]. Their mass spectra were also compared with known, previously obtained, compounds or from the Trace DSQ-MASS spectral database (Thermo).

### 3.10. Statistical Analysis

Statistical analysis of the experimental data points was performed by the ANOVA test, which was used for comparison of measured data using SPSS 12.0 statistical software (SPSS INC. Chicago, IL, USA). Differences were considered as statistically significant at *p* < 0.05.

## 4. Conclusions

This is the first report concerning the inhibitory effect of the essential oil isolated from the leaves of *V. negundo *Linn on melanin production. We also analyzed the chemical composition and antioxidant capacities of the essential oil. The present study concludes that *V. negundo *Linn essential oil shows antioxidant potential while simultaneously inhibiting melanin synthesis in B16F10 melanoma cells. The results indicated that *V. negundo* essential oil decreased melanin production and this may be attributed to its inhibitory action upon the signaling pathway regulating tyrosinase activity and/or depletion of cellular oxidative stress. The essential oil can thereby serve as a natural antioxidant which could also inhibit melanin production. Our research suggests that essential oils extracted from leaves of *V. negundo* Linn could be added to the cosmetic formulation of skin-whitening products. 
